# Fertility Preservation

**DOI:** 10.5935/1518-0557.20160022

**Published:** 2016

**Authors:** Paulo Franco Taitson, Antonio Mourthe Filho

**Affiliations:** 1 Pontifical Catholic University of Minas Gerais

**Keywords:** Fertility preservation, reproduction, oocyte vitrification

Gynecologic malignancies account for 1.09 million new cancer cases worldwide and for about 12% of the tumors affecting females. Approximately 10% of all female cancer survivors are aged 40 years or younger. Since cancers affecting female genital organs are usually treated with radical surgery, chemotherapy or chemoradiotherapy - approaches that induce permanent reproductive function impairment - the development of strategies to preserve fertility is one of the most important goals in gynecologic oncology (Tomao *et al*., 2016).

Fertility preservation is a hot topic in the field of reproduction. In addition to lifting the pressures of the biological clock, fertility preservation works as insurance for women seeking to get pregnant in later stages of their lives. Many are the reasons why a woman might wish to postpone pregnancy, including finding the right partner, pursuing education and career opportunities, or dealing with unexpected medical conditions. (Taitson *et al*., 2014)

In the past, cryopreservation of human oocytes was mainly unsuccessful due to the large water content of oocytes, which resulted in the formation of ice crystals within the oocytes. The ice crystals resulted in poor survival of the thawed oocytes. Now, with the advances of vitrification (ultra-rapid cooling), human oocytes can be cryopreserved with excellent survival and pregnancy rates. Research has proven that oocyte vitrification is safe and offers women a new way to preserve fertility. In 2012, the American Society for Reproductive Medicine (ASRM) stated that egg vitrification was no longer considered an experimental procedure (Andersen, 2015).

Oocyte vitrification is an elective medical procedure used to successfully cryopreserve human oocytes. The age at which patients usually undergo oocyte vitrification ranges from 21 to 39 years of age. In fertility preservation, the younger a woman is when she has her eggs vitrified, the better are her chances of having a successful pregnancy at a later time. Aging negatively impacts a woman's fertility. The decrease in fertility is largely explained by increased levels of aneuploidy (abnormal chromosomes) in human oocytes, which contributes to infertility, miscarriage and genetic anomalies in live born infants.

Fertility preservation processes are very similar to in-vitro fertilization. Female patints are screened prior to treatment to ensure they are good candidates for oocyte vitrification. Follicle-stimulating hormone (FSH) levels, antral follicle counts (AFC), and antimullerian hormone (AMH) levels are measured on day three of the menstrual cycle. Medical and special needs are addressed. The ovaries are stimulated with gonadotropin injections to support the development of multiple follicles, and ovulation is blocked with either a GnRH agonist or antagonist. The ovaries are usually stimulated for about 10 days, and follicular development is monitored with regular transvaginal ovarian ultrasound examination and blood work. Mature oocytes are then removed from the follicles by transvaginal ultrasound-guided oocyte aspiration. Good quality oocytes are vitrified and stored in liquid nitrogen (at -196 °C) for future use. Vitrified oocytes can be stored safely for many years.

Interestingly, a close friend told me that she offered to pay for the cryopreservation of the eggs of her daughters as a gift for their thirtieth birthdays. As a parent of a teenage daughter myself, this idea intrigued me and I thought long and hard about what new technology will be available when my daughter turns thirty. I feel confident that the field of fertility preservation is going to keep advancing in new and exciting ways, resulting in new procedures to help successfully beat the pressures of the biological clock.
